# Efficacy of Tocilizumab in Limbic Encephalitis with Anti-CASPR2 Antibodies

**DOI:** 10.1155/2020/5697670

**Published:** 2020-02-14

**Authors:** Maurizio Benucci, Luciana Tramacere, Maria Infantino, Mariangela Manfredi, Valentina Grossi, Arianna Damiani, Francesca Li Gobbi, Maristella Piccininni, Gaetano Zaccara, Massimo Cincotta

**Affiliations:** ^1^Rheumatology Unit, S. Giovanni di Dio Hospital, Florence, Italy; ^2^Neurology Unit, S. Giovanni di Dio Hospital, Florence, Italy; ^3^Immunology and Allergology Laboratory, S. Giovanni di Dio Hospital, Florence, Italy

## Abstract

We report the case of a 64-year-old man who presented with subacute memory, balance impairment, behavioral and mood changes, and epileptic seizures. Magnetic resonance imaging (MRI) showed bilateral hippocampal abnormalities. Brain [^18^F]-FDG fluorodeoxyglucose positron emission tomography (PET) revealed hypometabolism in both the temporal lobe as well as in the left insular and parietal regions. The clinical and neuroradiological picture and the detection of anti-CASPR2 antibodies in serum oriented the diagnosis towards autoimmune limbic encephalitis. Intravenous high-dose steroid and immunoglobulin treatments were ineffective. We did not use rituximab for the presence of antibodies to HbcAg positivity. Tocilizumab given intravenously 8 mg/kg once a month for six months and then subcutaneously 162 mg every week for six months resulted in clinical and neuroradiological improvement. These data support the efficacy of tocilizumab in autoimmune limbic encephalitis associated with anti-CASPR2 antibodies, which has been sporadically reported in the literature.

## 1. Introduction

Autoimmune etiology is increasingly recognized as a major cause of encephalitis [1–3]. The spectrum of autoimmune encephalitis (AE) is rapidly expanding with the continuous discovery of novel auto-antibodies [4]. Moreover, in other patients, clinical and magnetic resonance imaging (MRI) findings support the diagnosis of probable AE without demonstrated auto-antibody [5]. In a subset of patients, the outcomes following immunotherapies are good, regardless of the identification of auto-antibodies [6, 7]. For patients nonresponsive to first-line immunotherapy such as steroids and IVIg, second-line immunotherapies such as rituximab, which induces CD20+ B-cell destruction, may yield substantial benefit [8]. Tocilizumab is a humanized anti-interleukin (IL)-6 receptor monoclonal antibody, which blocks IL-6-mediated signal transduction [9]. Owing to the crucial role of IL-6 in stimulating both B and T cells in autoimmune processes [10–12], tocilizumab was established as efficient in various autoimmune diseases, including rheumatoid arthritis and systemic lupus erythematosus [13, 14]. Among the autoimmune central nervous system (CNS) disorders, a recent study reported a powerful disease modulation by tocilizumab in rituximab-refractory neuromyelitis optica (NMO) [15]. Regarding AE, successful treatment by tocilizumab was reported in several patients [16, 17], but in only one of these cases, contactin-associated protein-like 2 (CASPR2) antibodies were detected [16].

## 2. Case Report

A 64-year-old man was admitted to the neurology ward because of a disorder starting four months earlier with a one-week-long hypomanic episode. Subsequently, he had presented attention deficit, loss of recollection for recent events, and repetitive questioning, anxiety, loss of emotional control, balance disorder, and secondarily generalized seizures preceded by impaired language production.

The medical history consisted of hypertension, cervical spondylosis, and a previous, short episode of paroxysmal atrial fibrillation.

On admission, routine blood tests including erythrocyte sedimentation rate were normal. Also, blood serology for cytomegalovirus, herpes simplex 1 and 2, herpes zoster, human immunodeficiency (HIV) viruses, and Lyme disease were negative.

**Figure 1:**
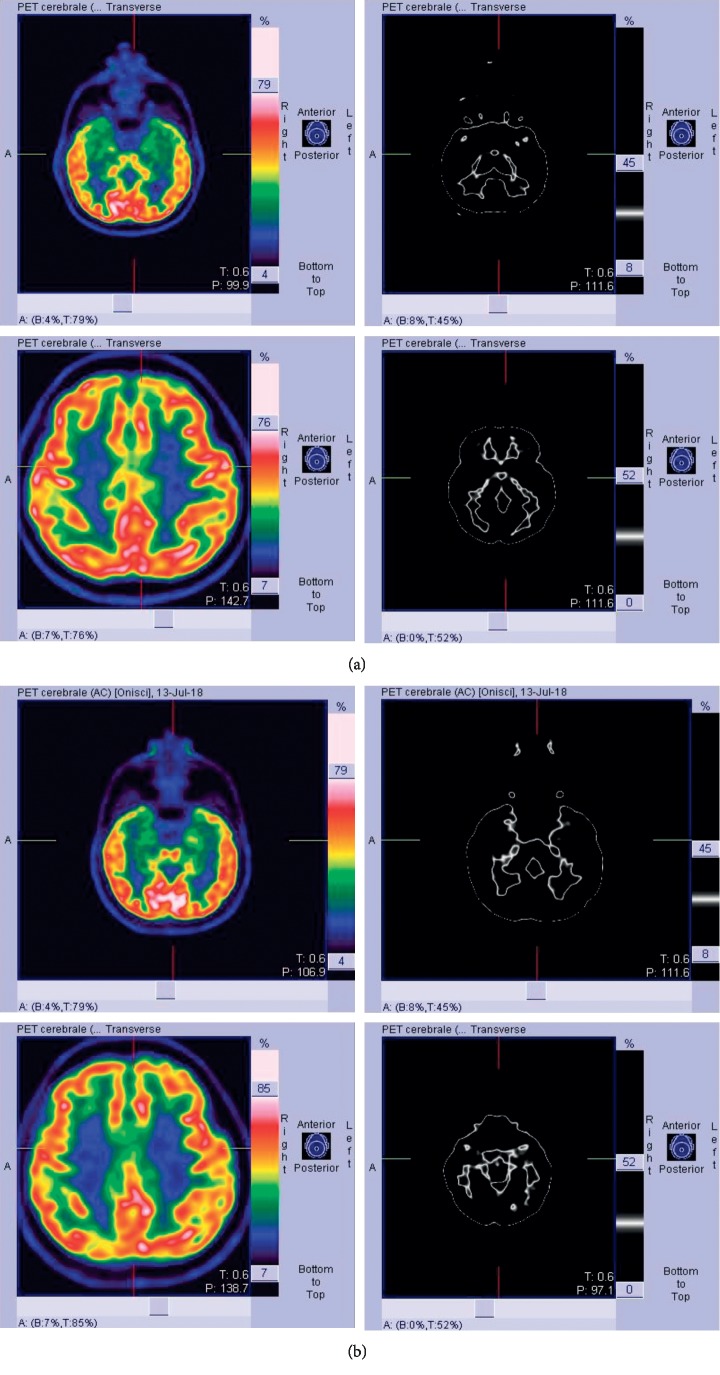
[^18^F] fluorodeoxyglucose positron emission tomography ([^18^F]-FDG PET) showed glucose metabolic alterations in both temporal lobes, mainly involving the mesial regions, and in the left insular and parietal regions.

**Figure 2:**
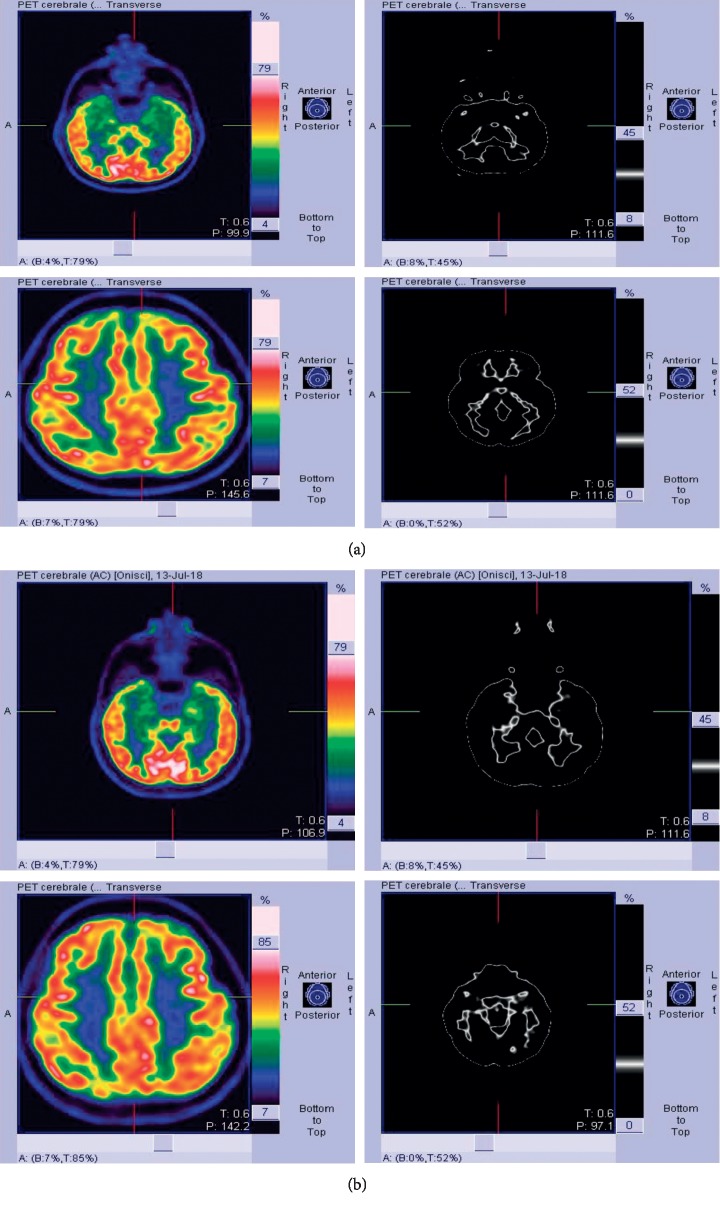
A significant improvement in metabolic uptake at [18F] fluorodeoxyglucose positron emission tomography ([18F]-FDG PET).

Anti-dsDNA antibodies, antiextractable nuclear antigens, antiphospholipid antibodies, and antineutrophil cytoplasmic antibodies were negative. Cerebrospinal fluid (CSF) examination showed slightly increased protein (0.57 g/l) and normal cellular content, as well as unremarkable IgG index. CSF oligoclonal bands were absent. No neurotrophic viruses neither mycobacterium tuberculosis were identified by CSF polymerase chain reaction analysis. Testing for anticontactin-associated protein 2 (CASPR2) auto-antibodies performed by indirect immunofluorescence on transfected cells and control-transfected cells (EU 90) at a starting dilution of 1 : 10 detected IgG against CASPR2 in serum (1 : 1000) (Euroimmun, Lübeck, Germany). We also evaluated the determination of anti-NMDA glutamate receptor antibodies, antileucine-rich glioma-inactivated 1 (LGI1), and antibody panels for paraneoplastic neurological syndromes with negative results. Antibody assessment was not performed in the cerebrospinal fluid. MRI showed slight bilateral hippocampal swelling, and mild FLAIR hyperintensity [^18^F] fluorodeoxyglucose positron emission tomography ([^18^F]-FDG PET) showed glucose metabolic alterations in both temporal lobes, mainly involving the mesial regions and in the left insular and parietal regions (Figure 1). Chest and abdomen CT scan, testicular ecography, and search for tumoral markers were negative. Clinical, neuroradiological, and laboratory investigations oriented the diagnosis towards anti-CASPR2 antibody-associated autoimmune limbic encephalitis. At the initiation of first-line immunotherapy, functional status assessed using the modified Rankin Scale (mRS) score was 3. High-dose steroid therapy (i.v. methylprednisolone 1 g daily for 5 days) and administration of IVIg 2 g/kg in 5 days were ineffective. Conversely, cognitive disturbances worsened and disinhibited behaviour appeared in spite of risperidone treatment. In addition, despite the intake of lacosamide 200 mg daily and levetiracetam 2000 mg daily, seizure frequency increased to 2-3 per month. We did not use rituximab for the presence of antibodies to HbcAg positivity (total Hb core positive) and anti-HBV antibodies 64.90 mU/ml. The patient presented a negative QuantiFERON (ESAT-6-CFP10 CD4/CD8). Flow cytometric analysis of lymphocyte profiles showed CD3+ 557 cells/mcl, CD4+ 386 cells/mcl, CD8+ 142 cells/mcl, CD56+ 111 cells/mcl, CD14HLADR+ 745 cells/mcl, CD28+ 35.9 cells/mc, CD28– 0.5 cells/mcl, CD19+ 184 cells/mcl, CD20+ 169 cells/mcl, CD38+ 8.4 cells/mcl, CD27+ naive 133 cells/mcl, and CD27+ memory 2 cells/mcl. The value of the circulating interleukin (IL)-6 was 23.9 pg/mL (human IL-6 instant enzyme-linked immunosorbent assay, eBioscience, Bender MedSystem GmbH, Vienna, Austria; normal value <3 pg/mL). We started tocilizumab 8 mg/kg once a month for six months i.v. and then 162 mg every week sc. This treatment determined cognitive, balance, and a significant improvement in metabolic uptake at [18F] fluorodeoxyglucose positron emission tomography ([18F]-FDG PET) (Figure 2). Findings of formal neuropsychological assessment before and after one month of tocilizumab treatment are shown in Table 1. Moreover, behavioral changes and seizures subsided. Also, the titer of anti-CASPR2 antibodies was also reduced to 1 : 100. After 4 months of therapy, functional status assessed using the mRS score was 0, and the patient was able to resume his work.

## 3. Discussion

In recent years, case reports and clinical series have been published in the use of tocilizumab in AE [17, 18]. Ninety-one patients with inadequate clinical response to first-line immunotherapy and following rituximab were retrospectively reviewed. Thirty (33.0%) patients were included in the tocilizumab group, 31 (34.0%) in the additional rituximab group, and 30 (33.0%) in the observation group. The tocilizumab group showed more frequent favorable mRS scores at 2 months from treatment initiation and at the last follow-up compared with those at the relevant time points of the remaining groups. The majority (89.5%) of the patients with clinical improvement at 1 month from tocilizumab treatment maintained a long-term favorable clinical response [17]. According to the auto-antibody tests of the patients' serum/CSF, 26 (28.6%) patients had anti-NMDA-R antibodies, 3 (3.3%) had antileucine-rich glioma-inactivated 1 antibodies, and 2 (2.2%) had antiamphiphysin antibodies. The remaining 60 (65.9%) patients showed negative results on the auto-antibody detection test, and no patient had anti-CASPR2 antibodies [17]. In another clinical series on 3 cases, tocilizumab has also been used successfully in pediatric forms [19]. Therefore, the drug was included as a third line of treatment even in the encephalitis forms of children [20]. Based on the review of the literature, this is the second case of autoimmune encephalitis with antipositive CASPR2 treated with tocilizumab [16]. In our patient, the therapy resulted in a titer reduction of anti-CASPR2 antibodies although this does not seem to correlate according to some reports with the prognostic trend [21]. The presence of CASPR2 antibodies is associated with a subgroup of autoimmune-mediated neurological disorders, including limbic encephalitis, neuromyotonia, and Morvan syndrome [22]. A previous study has revealed the efficacy of tocilizumab in rituximab-refractory NMO spectrum disorders [15]. The efficacy of tocilizumab can be explained by the extensive pathophysiologic mechanisms by which IL-6 induces and enhances autoimmunity [23, 24]. IL-6 not only induces B-cell differentiation and proliferation but also promotes the differentiation of a main inducer of autoimmune tissue damage, IL-17-producing T-helper cells, from naive T cells [24]. IL-6 inhibits regulatory T-cell differentiation, which is crucial in maintaining the balance against IL-17-producing T-helper cells in regulating autoimmune processes. Furthermore, IL-6 stimulates the differentiation of CD8+ cytotoxic T cells, which promote excitotoxicity-induced neuronal damage [25]. Recently, the safety of tocilizumab has also been observed in demyelinating pathologies and CNS complications in rheumatoid arthritis [26].

Our clinical case confirms that tocilizumab can improve the cases of autoimmune encephalitis with anti-CASPR2 antibodies, and this therapeutic strategy can be reserved for patients resistant or intolerant to other first- or second-line immunosuppressive therapy.

## Figures and Tables

**Table 1:** Evaluation of psychometric tests during tocilizumab treatment.

Psychometric tests	First evaluation score	Second evaluation score
Rey auditory verbal learning test	0	2
Babcock story recall test	4	4
Semantic verbal fluency test	4	4
Phonemic verbal fluency test	3	4
Attentive matrices test	2	4
Trail making test	0	2
